# Genomic breeding value prediction using three Bayesian methods and application to reduced density marker panels

**DOI:** 10.1186/1753-6561-4-S1-S6

**Published:** 2010-03-31

**Authors:** Matthew A Cleveland, Selma Forni, Nader Deeb, Christian Maltecca

**Affiliations:** 1 Genus plc., 100 Bluegrass Commons Blvd., Suite 2200, Hendersonville, TN, 37075, USA; 2 North Carolina State University, Department of Animal Science, Raleigh, NC, 27695-7627, USA

## Abstract

**Background:**

Bayesian approaches for predicting genomic breeding values (GEBV) have been proposed that allow for different variances for individual markers resulting in a shrinkage procedure that uses prior information to coerce negligible effects towards zero. These approaches have generally assumed application to high-density genotype data on all individuals, which may not be the case in practice. In this study, three approaches were compared for their predictive power in computing GEBV when training at high SNP marker density and predicting at high or low densities: the well- known *Bayes-A,* a generalization of *Bayes-A* where scale and degrees of freedom are estimated from the data (*Student-t*) and a Bayesian implementation of the* Lasso* method. Twelve scenarios were evaluated for predicting GEBV using low-density marker subsets, including selection of SNP based on genome spacing or size of additive effect and the inclusion of unknown genotype information in the form of genotype probabilities from pedigree and genotyped ancestors.

**Results:**

The GEBV accuracy (calculated as correlation between GEBV and traditional breeding values) was highest for* Lasso,* followed by* Student-t* and then* Bayes-A.* When comparing GEBV to true breeding values,* Student-t* was most accurate, though differences were small. In general the shrinkage applied by the* Lasso* approach was less conservative than* Bayes-A* or* Student-t,* indicating that* Lasso* may be more sensitive to QTL with small effects. In the reduced-density marker subsets the ranking of the methods was generally consistent. Overall, low-density, evenly-spaced SNPs did a poor job of predicting GEBV, but SNPs selected based on additive effect size yielded accuracies similar to those at high density, even when coverage was low. The inclusion of genotype probabilities to the evenly-spaced subsets showed promising increases in accuracy and may be more useful in cases where many QTL of small effect are expected.

**Conclusions:**

In this dataset the* Student-t* approach slightly outperformed the other methods when predicting GEBV at both high and low density, but the* Lasso* method may have particular advantages in situations where many small QTL are expected. When markers were selected at low density based on genome spacing, the inclusion of genotype probabilities increased GEBV accuracy which would allow a single low- density marker panel to be used across traits.

## Background

A number of approaches have recently been proposed for the prediction of genomic breeding values for high-density single nucleotide polymorphism (SNP) panels. Methods commonly used fall into two categories,* BLUP* and Bayesian approaches. In a* BLUP* framework SNP effects are sampled from a normal distribution and the variance is assumed constant across SNPs [1]. In a Bayesian approach prior knowledge about the distribution of SNP effects is assumed, generally that many SNPs are likely to have small individual effects and only a few will have large effects [2], allowing for different variances for individual SNPs. This assumption results in a shrinkage procedure in which the prior information is used to coerce negligible effects toward zero. Different derivations of this shrinkage approach have been proposed, including * Bayes-A*[1]. In this method a scaled inverse-χ^2^ prior is assigned to SNP variances. Scale and degrees of freedom of the distribution are in this case set as hyperparameters and samples of the posterior distribution are obtained through MCMC methods. A generalization in which the hyperparameters regulating the shrinkage are treated as unknown parameters and estimated from the data leads to the well known* Student-t* model [3] where the amount of shrinkage is controlled by the data. Alternative shrinkage approaches have also been recently proposed. A particularly appealing method is the least absolute shrinkage and selection operator (*Lasso*) [4]. In its Bayesian interpretation* Lasso* estimates can be seen as posterior mode estimates when the regression parameters have independent and identical Laplace priors. Yi and Xu [5] recently compared* Lasso* and* Student-t* models for QTL mapping. Prediction of genomic breeding values can be seen as a generalization of the same problem. It has been reported [6,7] that Bayesian methods give higher genomic breeding value accuracies than* BLUP* methods. There are few published results, though, on the performance of different shrinkage methods for genomic breeding value prediction. These approaches were initially developed assuming dense genome- wide SNP coverage. This may not be the case in practice as it is often cost prohibitive to genotype all animals at high density and it may be desired to predict genomic breeding values using low density panels.

This study investigated the predictive performance of different Bayesian hierarchical approaches,* Bayes-A, Student-t* and *Lasso,* when training and predicting genomic breeding values at high density and when predicting at lower densities.

## Methods

The dataset used for analysis was simulated as part of the 13^th^ QTL-MAS Workshop, see [8] for details. The data consisted of 5 sires, 20 dams and 2000 offspring, of which 1000 had phenotypes. The 1000 phenotyped offspring made up the training set, while the 1000 un-phenotyped offspring comprised the prediction set for calculation of genomic breeding values (GEBV). All individuals were genotyped for 453 SNP markers, approximately equally-spaced across five chromosomes of length one Morgan each.

### Prediction of phenotypes and breeding values

The simulated dataset included phenotypes for five traits representing measures of yield at five different time points (t0, t132, t265, t397 and t530). A sixth phenotype was predicted to represent yield at a time point beyond the simulated data, time point 600 (t600). A number of non-linear models were tested to predict t600 [9-12], and the Gompertz model [12] was found to best fit the data according to AIC [13] and BIC [14] measures. Least squares estimates of growth curve parameters were obtained for each individual using the procedure "NLIN" from SAS [15]. Individual growth curve parameters could then be used to calculate individual phenotypic predictions for any time point until maturity, including t600. Traditional breeding values were estimated using a single trait linear model for each of the time points. We report the results for t530 and t600.

### Description of models

The data were analyzed using three different approaches, considering additive genetic effects only. The general structure of the models in matrix form was:

where **y** is the vector of phenotypic effects, **µ** is the overall mean, ***β*** is the vector of additive effects for each marker,***X*** is a matrix of genotypes expressed as number of copies of an arbitrary allele (0,1,2) for each SNP and **e** is a vector of residuals assumed N(0,). All models were considered as two level hierarchical models. A flat (1) and a non informative (1/) prior were assigned to µ and , respectively. The remaining prior structure was:

for the j^th^ SNP,

for the* Lasso* approach and

for the* Bayes-A* and* Student-t* approaches. Degrees of freedom* v* and scale parameter *s^2^* for* Bayes-A* were considered hyperparameters and were assigned values as in [1]. The* Student-t* model treated* v* and* s^2^* as unknown and assigned a uniform density of 1/*v* for the interval (0,1] and a uniform distribution of *s* for the range (0,A], with A being a large number [5]. The* λ* parameter in the* Lasso* approach was assigned a gamma prior distribution *Gamma*(*a*, *b*). Values of* a* and* b* were set at 0.05 and 1.0, respectively, so that prior of *λ* was essentially uniform over a wide range of values. The* Lasso* approach differs slightly from that of Park and Casella [16] and de los Campos [17], which is guaranteed to provide unimodal posteriors of effects. A Gibbs sampling algorithm was implemented to obtain samples from the joint posterior distribution. Steps of the algorithm are outlined below (for details on conditional posterior distribution see [5]):

*1) Sample µ from N*(*µ* | *y*, *β*,)

*2) Sample β_j_ from N*(*β* | *y*,* µ*,,), *where the updates in this case are obtained though Gauss-Seidel with residual update *[18]

*3) Sample**from Inv* − χ^2^ (|* y*,*µ*,*β*)

4) i. Lasso method:

*Sample*. *from InvGauss*(|* β_j_* ,*λ*)

*Sample λ from Gamma*(*λ*^2^ | )

ii. Bayes-A, Student-t:

*Sample**from Inv −  χ^2^*( | *β,v,s^2^*) 

For Student-t only:

*Sample s^2^ from Gamma* (*s^2^* | ,, *v*)

*Sample v with a Metropolis step* (*v* | *s*^2^, )

5) return to Step 1

The Gibbs sampling algorithm for all three methods was implemented in R [19]. For each analysis a single chain of 15000 iterations was run with a burn-in period of 5500 iterations. Samples were stored every 30 iterations. Convergence of each chain was assessed both by visual inspection of the trace and the use of estimates of effective sample size for the variances obtained through the R coda package [20]. Inferences on the parameters were made on the average of the posterior samples after burn-in.

Genomic breeding values were calculated for all individuals in the prediction set, for t530 and t600 by:

where ***X_m_*** is a matrix of genotypes expressed as (0,1,2) and ***β_m_*** is a vector of posterior mean effects for a particular method, for* m* SNPs. A cross validation procedure was also used where phenotyped individuals were randomly split into training and prediction sets (90% training; 10% prediction) 10 times to assess the stability of the genomic predictions for t530 and t600.

### Low-density marker subsets

Subsets of the prediction dataset were created to simulate the situation where training can be done at high density, but prediction of GEBV occurs with a lower density panel. In this case the full training set, including 1000 individuals and 453 SNPs, was used to estimate the SNP effects, but GEBV were calculated using either a smaller subset of SNPs or a combination of genotypes for a small subset and genotype probabilities for the remaining markers (see Table 1). Genomic breeding values were calculated for 12 subset scenarios without retraining on the subset markers, using each of the three methods, for t530 and t600. One-half of the data subsets included only a small number of markers spaced evenly across the genome (*m*=19, 38 or 76) or a small number of markers with the largest absolute effects in each of the respective methods and traits (*m*=19, 38 or 76). These SNP numbers were chosen to approximate the low-density panels that could potentially be used in livestock species (e.g., 384 in pigs or cattle). These GEBV calculations used effects only for these (*m*) markers from the training set. For each of the subsets above another subset was tested including genotype probabilities for all of the remaining markers in calculation of the GEBV. Each of these subsets contained all markers, and thus these GEBV calculations used all high-density SNP effects from training, but only a small subset of markers had actual genotypes (*m*=19, 38 or 76). The genotype probabilities were calculated through marker and pedigree information from the full dataset, for all individuals in the prediction set, using segregation analysis for single markers following Kerr and Kinghorn [21].

**Table 1:** Number of SNPs included in the calculation of genomic breeding values in each low-density scenario

Scenario	Evenly-spaced^a^	Largest effects^b^	Genotype	Total
			probabilities^c^	
EVEN_19	19			19

EVEN_38	38			38

EVEN_76	76			76

SIG_19		19		19

SIG_38		38		38

SIG_76		76		76

EVEN_GP_19	19		434	453

EVEN_GP_38	38		415	453

EVEN_GP_76	76		377	453

SIG_GP_19		19	434	453

SIG_GP_38		38	415	453

SIG_GP_76		76	377	453

^a^Selected by taking the *m*^th^ SNPs from ordered list and thus SNP were approximately evenly-spaced

^b^Selected by taking the top *m* SNPs from a list ordered by absolute effect, for each analysis method

^c^Genotype probabilities were used in place of actual genotypes for all SNPs that don't fall into one of the other categories, within a scenario

Genomic breeding values were calculated for the marker subsets as above by:

In this case, the individual element (*i*) of *** X_m_*** is calculated as:

where* P(1)* and* P(2)* are the probabilities of individual (*i*) having the heterozygous and homozygous (coded as 2) genotypes, respectively, for each marker (*j*). When the actual genotype is known the matrix element is simply coded as before (0,1,2). This approach is related to the genetic predictor approach of Boer* et al.*[22].

## Results

Accuracies of the GEBV were calculated for each of the three approaches (*Bayes-A, Student-t* and* Lasso*) as the correlation between the GEBV and estimated breeding values (EBV) calculated using the traditional animal model, for all animals in the prediction set (Table 2). The three approaches performed similarly with* Lasso* yielding the highest accuracy, followed by* Student-t* and then* Bayes-A.* The difference between the top and bottom accuracies was about 6%. Results were consistent across t530 and t600. Coefficients of regression of EBV on GEBV were nearly identical for all methods, across traits, indicating little or no difference in bias exists. Cross- validation using ten replicates from the training dataset found differences between the three approaches consistent with Table 2 (results not shown).

**Table 2:** Correlations between genomic breeding values and breeding values from a traditional animal model for animals in the prediction set (without phenotypes) and coefficients of regression of traditional on genomic breeding values, for t530 and t600.

		t530		t600
**Method**	**Corr.**	**b**	**Corr.**	**B**

*Bayes-A*	0.673	0.893	0.674	0.880

*Student-t*	0.718	1.019	0.720	1.010

*Lasso*	0.736	1.061	0.737	1.072

Correlations of GEBV for each low-density SNP scenario (Table 1) and EBV (including the change in correlation compared to GEBV calculated using all markers in the prediction set) are shown in Table 3, to represent the change in GEBV accuracy using the low-density approach. In all cases the accuracy increased (or stayed the same) when increasing the number of markers with genotypes in the subset. The scenarios where evenly-spaced markers were included had lower accuracies than the same density subset where SNPs with the largest effects were included. There appears to be an advantage to using genotype probabilities with evenly-spaced markers, particularly in the case with few marker genotypes (EVEN_19) with accuracies approaching 1. The differences between the three models were similar to those found when using all markers in the prediction set (Table 2), but the reductions in accuracy using* Bayes-A* were the smallest in nearly all cases. When using SNPs with the largest effects (with and without genotype probabilities), the GEBV calculated using* Bayes-A* were essentially the same as GEBV calculated using all markers. There was little loss in accuracy by reducing the marker set from 453 to 19 (with the largest effects) for all methods. The reductions in accuracy resulting from* Lasso* marker effects were generally similar to the other methods for all low-density subsets and the accuracy was still superior to* Bayes-A* and* Student-t* in all cases. In a number of the scenarios the accuracy actually increased from high-density to low-density. Many of the increases were small and thus the accuracies were practically unchanged, but large increases in accuracy were observed for subsets with evenly-spaced SNPs and genotype probabilities, particularly EVEN_GP_19.

**Table 3:** Correlations between genomic breeding values and breeding values from different low SNP-density approaches (and change in correlation compared to original full marker model), where all SNP effects are estimated in the same high SNP-density training set, for t530 and t600.

		t530			t600	
**Scenario**	***Bayes-A***	***Student-t***	***Lasso***	***Bayes-A***	***Student-t***	***Lasso***

EVEN_19	0.255	0.142	0.195	-0.128	0.098	0.173
	(-0.418)	(-0.846)	(-0.594)	(-0.532)	(-0.622)	(-0.564)

EVEN_38	0.481	0.494	0.528	0.469	0.485	0.522
	(-0.192)	(-0.249)	(-0.242)	(-0.180)	(-0.235)	(-0.215)

EVEN_76	0.490	0.544	0.586	0.472	0.532	0.584
	(-0.183)	(-0.246)	(-0.192)	(-0.130)	(-0.188)	(-0.153)

SIG_19	0.663	0.699	0.709	0.669	0.692	0.709
	(-0.010)	(-0.049)	(-0.037)	(0.025)	(-0.028)	(-0.028)

SIG_38	0.664	0.703	0.713	0.669	0.707	0.721
	(-0.009)	(-0.049)	(-0.033)	(0.029)	(-0.013)	(-0.016)

SIG_76	0.667	0.709	0.711	0.672	0.712	0.729
	(-0.006)	(-0.046)	(-0.027)	(0.035)	(-0.008)	(-0.008)

EVEN_GP_19	0.937	0.967	0.980	0.928	0.967	0.978
	(0.264)	(0.210)	(0.231)	(0.293)	(0.247)	(0.241)

EVEN_GP_38	0.733	0.785	0.861	0.736	0.789	0.862
	(0.060)	(0.018)	(-0.049)	(0.111)	(0.069)	(0.125)

EVEN_GP_76	0.733	0.786	0.854	0.736	0.789	0.856
	(0.060)	(0.018)	(-0.050)	(0.112)	(0.069)	(0.119)

SIG_GP_19	0.674	0.730	0.802	0.675	0.735	0.798
	(0.001)	(0.043)	(-0.006)	(0.056)	(0.015)	(0.061)

SIG_GP_38	0.673	0.728	0.783	0.675	0.731	0.791
	(0)	(-0.043)	(-0.008)	(0.054)	(0.011)	(0.054)

SIG_GP_76	0.673	0.724	0.767	0.674	0.729	0.769
	(0)	(-0.044)	(-0.012)	(0.050)	(0.009)	(0.032)

## Discussion

**Figure 1:**
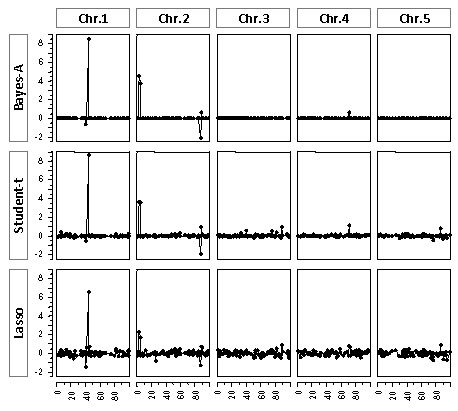
**SNP effects estimated** by* Bayes-A, Student-t* and* Lasso* for t600, by genome location (cM).

The three methods applied to the simulated data performed similarly (Table 2), where the accuracy using* Lasso* was the highest,* Student-t* was next and then* Bayes-A,* though differences were small. The accuracy in this case was the correlation between GEBV and EBV, which is the limit of information currently available, and thus the reported accuracies will likely change when true breeding values are available. In general, methods based on* inverse -χ^2^* priors (*Bayes-A* and* Student-t*) appear to be more conservative in the shrinkage than* Lasso,* even when the scale and degrees of freedom parameters are estimated from the data (Figure 1). These parameters estimated by* Student-t* and* Lasso* all converged to the same values (within method) across the cross-validation replicates, indicating that this dataset included sufficient information for estimation. The marker effects shown in Figure 1 suggest that* Lasso* may be more sensitive to QTL with small effects than* Student-t,* which in turn is more sensitive than *Bayes-A*.

The use of low-density SNP subsets is based on the concept of Habier* et al.*[23] where SNP effects are estimated from a training dataset using high-density SNP genotypes, but GEBV are then calculated for individuals genotyped for only a small subset of the SNPs. These subsets may be chosen by selecting markers for even genome coverage or based on effect size for a certain trait, where un-genotyped SNPs may be filled in to approximate high-density coverage. The current analysis found that evenly-spaced SNPs alone did a poor job of predicting GEBV (Table 3). By chance this approach could produce high GEBV accuracies if selected SNPs happened to be in linkage disequilibrium (LD) with large QTL for a particular trait, but in general it would be expected that many QTL would not be represented by the low-density panel. In the current dataset average LD was low (results not shown) which explains the poorer performance of the evenly-spaced, low-density subset compared to other approaches. Selecting only SNPs with large effects in each of the three methods yielded GEBV that were nearly as accurate as when using all markers, in all cases. This result is likely specific to the case where few QTL of large or moderate effect are expected and thus few markers will account for most of the variance, which is presumed in this dataset based on Figure 1. In fact, the correlation between GEBV and EBV for t600 in the prediction set was 0.603 using the three SNPs with largest effects in* Bayes-A,* only a 7% reduction in accuracy.

The scenarios using genotype probabilities performed well and in most cases showed a small or no reduction in accuracy, compared to using the full marker set. Due to the population structure (full and half-sib families) and completeness of parental genotypes it is expected that the genotype probabilities are a good representation of the true genotypes in this case. In a situation where there are fewer ties between individuals the advantage of using genotype probabilities (in place of actual genotypes) is likely to be lower than what was found in this study. A number of the scenarios even showed large increases in accuracy to unrealistic levels (e.g., EVEN_GP_19, Table 3). Paradoxically, the evenly-spaced scenarios outperformed the largest SNP effects scenarios, where the best performance came from the smallest number of SNPs. This result can be attributed to calculating accuracy based on the EBV. With fewer markers and less information (based on even spacing) the GEBV calculated in EVEN_GP_19 are nearly identical within family and are implicitly based on family relationships, through SNP allele sharing, and thus the GEBV are approximations of the EBV rather than the true breeding value. Using the EBV as a proxy for the true breeding value appears to be a poor choice in this case. Addition of true breeding values should make this a fairer comparison.

## Epilogue

The availability of true breeding values (TBV) allowed for an improved evaluation of the effectiveness of the three analysis methods on alternative marker sets (Table 4). As expected, the correlations improved when comparing GEBV to TBV, instead of EBV. The accuracy of each of the methods was high when using all markers, with* Student-t* yielding the highest value (0.945). The differences between methods were small and more work is needed to determine if they are meaningful in practice. The* Student-t* method also had the highest accuracy for nearly all of the low- density SNP scenarios, though again the differences in these cases were small. Scenarios where markers were evenly-spaced had lower accuracies than when markers were selected based on effect size (EVEN versus SIG) due to the presence of few moderate or large QTL, but increasing from 19 to just 38 evenly-spaced SNPs was enough to yield accuracies greater than 0.70. There was a large increase in accuracy when including genotype probabilities in place of known genotypes for evenly-spaced SNPs, particularly when only 19 SNPs with actual genotypes were used. The comparisons with individual TBV also showed the expected decreases in accuracy with decreasing number of actual genotypes in the EVEN_GP scenarios that were not seen when comparing to EBV. Comparing the GEBV to the family mean TBV resulted in the paradoxical increase in accuracy as with the original GEBV/EBV comparison when using fewer markers in the EVEN_GP scenarios (results not shown). This highlights the need for care when using EBV for GEBV evaluation, in combination with genotype probabilities, in data with full-sib families. For this trait, though, including a small number of SNPs with large effects would be enough to obtain high accuracy GEBV while greatly reducing genotyping requirements. The results from using genotype probabilities are promising but are likely best applied in situations where many small QTL are expected.

**Table 4:** Accuracy of genomic breeding values using three methods, as the correlation between true and predicted breeding values, for animals in the prediction set using all markers (ALL) and using alternative low-density approaches, for t600.

Scenario	*Bayes-A*	*Student-t*	*Lasso*
ALL	0.916	0.945	0.916

EVEN_19	0.040	0.206	0.258

EVEN_38	0.732	0.738	0.738

EVEN_76	0.734	0.761	0.758

SIG_19	0.913	0.931	0.910

SIG_38	0.915	0.938	0.914

SIG_76	0.915	0.943	0.921

EVEN_GP_19	0.658	0.674	0.671

EVEN_GP_38	0.833	0.84	0.817

EVEN_GP_76	0.834	0.846	0.825

SIG_GP_19	0.914	0.937	0.914

SIG_GP_38	0.915	0.940	0.917

SIG_GP_76	0.916	0.943	0.920

## Conclusions

For this simulated dataset the* Lasso* method slightly outperformed* Bayes-A* and* Student-t* when considering accuracy as the correlation between GEBV and EBV, but* Student-t* performed the best when comparing GEBV to TBV.* Bayes-A* and *Student-t* appeared to be more conservative in shrinkage of SNP effects indicating that *Lasso* may be more sensitive to small QTL and thus may perform better than other methods for traits where large or moderate QTL are not expected. In the analysis of reduced marker density few SNPs were needed to maintain levels of accuracy similar to the high-density SNP set when SNPs with large effect were selected. When markers were selected based on spacing, the use of genotype probabilities in place of known genotypes increased the accuracy of the GEBV, which would allow a single low-density panel to be used across traits.

## Competing interests

The authors declare that they have no competing interests.

## Authors' contributions

MAC performed analyses, participated in study design and drafted the manuscript. SF performed analyses and participated in study design. ND participated in study design and helped to interpret results. CM developed the methods for effect estimation, performed analyses, participated in study design and helped draft the manuscript. All authors read and approved the final manuscript.

## References

[B1] Meuwissen THE Hayes BJ Goddard ME Prediction of total genetic value using genome-wide dense marker maps. Genet 2001 157 1819 1829 10.1093/genetics/157.4.1819 PMC1461589 11290733

[B2] Hayes BJ Goddard ME The distribution of the effects of genes affecting quantitative traits in livestock. Genet 2001 33 209 229 10.1186/1297-9686-33-3-209 11403745 PMC2705405

[B3] Andrews DF Mallows CL Scale mixtures of normal distributions. J Royal Stat Soc B-Methodological 1974 36 99 102

[B4] Tibshirani R Regression shrinkage and selection via the lasso. J Royal Stat Soc B 1996 58 267 288

[B5] Yi N Xu S Bayesian LASSO for quantitative trait loci mapping. Genet 2008 179 1045 1055 10.1534/genetics.107.085589 PMC2429858 18505874

[B6] VanRaden PM Efficient methods to compute genomic predictions. J Dairy Sci 2008 91 4414 4423 10.3168/jds.2007-0980 18946147

[B7] VanRaden PM Van Tassell CP Wiggans GR Sonstegard TS Schnabel RD Taylor JF Schenkel FS Invited review: Reliability of genomic predictions for North American Holstein bull. J Dairy Sci 2009 92 16 24 10.3168/jds.2008-1514 19109259

[B8] Coster A Bastiaansen J Calus M Maliepaard C Bink M QTLMAS 2009: Simulated dataset. BMCProc 2010 4 Suppl 1 S3 10.1186/1753-6561-4-S1-S3 PMC2857845 20380757

[B9] Brody S Bioenergetics and growth. 1945 Reinhold Publishing Corp.

[B10] Von Bertalanffy L Quantitative laws in metabolism and growth. The Quarterly Review of Biology 1957 32 217 230 10.1086/401873 13485376

[B11] Nelder JA The fitting of a generalization of the logistic curve. Biometrics 1961 17 89 110 10.2307/2527498

[B12] Laird AK Dynamics of relative growth. Growth 1965 29 249 263 5865687

[B13] Akaike H A new look at the statistical model identification. IEEE Trans Autom Control 1974 19 716 723 10.1109/TAC.1974.1100705

[B14] Schwarz G Estimating the dimension of a model. Annals of Stat 1978 6 8 461 464 10.1214/aos/1176344136

[B15] SAS Institute Inc SAS 9.2 Help and Documentation. 2009

[B16] Park T Casella G The Bayesian Lasso. JAmer Stat Soc 2008 103 681 686

[B17] de los Campos G Naya H Gianola D Crossa J Legarra A Manfredi E Weigel K Cotes JM Predicting quantitative traits with regression models for dense molecular markers and pedigree. Genet 2009 182 375 385 10.1534/genetics.109.101501 PMC2674834 19293140

[B18] Legarra A Misztal I Technical Note: Computing strategies in genome-wide selection. J Dairy Sci 2008 91 360 366 10.3168/jds.2007-0403 18096959

[B19] R Development Core Team R: A language and environment for statisitcal computing. 2008

[B20] Plummer M Best N Cowles K Vines K CODE: Convergence diagnosis and output analysis for MCMC. R News 2006 6 7 11

[B21] Kerr RJ Kinghorn BP An efficient algorithm for segregation analysis in large populations. JAnim Breed Genet 1996 113 457 469

[B22] Boer MP Wright D Feng L Podlich DW Luo L Cooper M van Eeuwijk FA A mixed-model quantitative trait loci (QTL) analysis for multiple- environment trial data using environmental covariables for QTL-by- environment interactions, with an example in Maize. Genet 2007 177 1801 1813 10.1534/genetics.107.071068 PMC2147942 17947443

[B23] Habier D Fernando RL Dekkers JCM Genomic selection using low-density marker panels. Genet 2009 182 343 353 10.1534/genetics.108.100289 PMC2674831 19299339

